# Antineoplastic effect of block-and-replace strategy for managing hypercortisolism caused by adrenal cortical carcinoma

**DOI:** 10.1210/jcemcr/luag262

**Published:** 2026-09-25

**Authors:** Davide Lorenzo Bettini, Sara Rodella, Marta Laganà, Antonella Turla, Alfredo Berruti, Deborah Cosentini

**Affiliations:** Medical Oncology, University of Brescia, ASST Spedali Civili Comprehensive Cancer Center, Brescia 25123, Italy; Medical Oncology, University of Brescia, ASST Spedali Civili Comprehensive Cancer Center, Brescia 25123, Italy; Medical Oncology, University of Brescia, ASST Spedali Civili Comprehensive Cancer Center, Brescia 25123, Italy; Medical Oncology, University of Brescia, ASST Spedali Civili Comprehensive Cancer Center, Brescia 25123, Italy; Medical Oncology, University of Brescia, ASST Spedali Civili Comprehensive Cancer Center, Brescia 25123, Italy; Medical Oncology, University of Brescia, ASST Spedali Civili Comprehensive Cancer Center, Brescia 25123, Italy

**Keywords:** adrenocortical carcinoma, Cushing syndrome, osilodrostat, metyrapone

## Abstract

Adrenocortical carcinoma (ACC) is a rare and aggressive malignancy frequently associated with cortisol hypersecretion, which confers substantial morbidity and a worse prognosis. In metastatic ACC, rapid control of Cushing syndrome is essential to allow systemic therapy. Although the block-and-replace strategy using steroidogenesis inhibitors is well established for cortisol control, its potential antineoplastic effects have not been previously reported. We describe 2 patients with metastatic ACC and severe Cushing syndrome treated with block-and-replace using osilodrostat or metyrapone. In both, cortisol normalized rapidly and safely, with marked clinical improvement. Unexpectedly, both experienced significant radiological tumor responses before exposure to effective cytotoxic chemotherapy and before therapeutic mitotane plasma concentrations were achieved. These cases suggest that steroidogenesis inhibition within a block-and-replace strategy may exert a direct or indirect antineoplastic effect in ACC. Although causality cannot be established, the temporal association between cortisol blockade and tumor response is compelling and warrants further prospective and translational investigation.

## Introduction

Adrenocortical carcinoma (ACC) is a rare and aggressive malignancy [1-3], and surgery is the mainstay of therapy. Management of metastatic disease not amenable to surgery is challenging. The goal is to control tumor growth and the associated hormone hypersecretion, particularly cortisol, which occurs in approximately 50% to 70% of patients and is associated with a worse prognosis [4, 5].

ACC-induced Cushing syndrome can be controlled with the block-and-replace strategy, which completely blocks endogenous cortisol production using potent adrenal steroidogenesis inhibitors and replaces physiologic glucocorticoid levels with exogenous supplementation [6-8].

Osilodrostat and metyrapone are potent oral 11-beta-hydroxylase inhibitors that block glucocorticoid biosynthesis [9, 10]. They swiftly restore urinary free cortisol excretion [9-12] and are the most effective drugs for implementing the block-and-replace strategy [6-8].

This strategy controls glucocorticoid excess and allows quick initiation of standard chemotherapy (etoposide, doxorubicin, cisplatin, and mitotane) [13].

Rapid cortisol inhibition could also have an antineoplastic effect, as cortisol excess has been hypothesized to promote ACC growth [14, 15]. Moreover, osilodrostat can cause adrenal gland shrinkage in ACTH-dependent Cushing syndrome, suggesting a potential direct or indirect adrenolytic effect [16].

We present evidence of a potential antineoplastic effect of this strategy in 2 patients with ACC.

## Case presentation

### Case 1

In February 2023, a 67-year-old woman presented to her general practitioner with recent-onset marked asthenia, hirsutism, flushing, and poor blood pressure control.

### Case 2

In June 2023, a 74-year-old man was admitted to another hospital with anasarca, diffuse ecchymosis, lower-limb edema, significant weight gain, and uncontrolled hypertension.

A left adrenal gland lesion (70 × 70 mm) was documented on computed tomography (CT) and 18F-fluorodeoxyglucose positron emission tomography/CT, with elevated plasma cortisol and suppressed ACTH. In November 2023, he underwent laparoscopic left adrenalectomy; histology confirmed ACC with a Ki67 index of 8% and a Weiss score of 4 (modified European Network for the Study of Adrenal Tumors stage II). No adjuvant therapy was given.

## Diagnostic assessment

### Case 1

Abdominal CT documented a large left adrenal mass (129 × 99 mm) infiltrating the peritoneum and surrounding organs (modified European Network for the Study of Adrenal Tumors stage IV). As the lesion was unresectable, biopsy provided a definitive ACC diagnosis. Ki67 proliferation index was 20%, and the Weiss score was 7.

She was referred to our center on March 27, 2023, 1 month after diagnosis. On admission, her general condition was poor, with an Eastern Cooperative Oncology Group (ECOG) performance status (PS) of 4; she was bedridden, with severe sarcopenia, mycotic mucositis, oral thrush, genital candidiasis, marked lower-limb edema, and uncontrolled hypertension despite beta-blocker, calcium antagonist, and angiotensin-converting enzyme inhibitor therapy. Blood tests revealed suppressed ACTH, elevated serum cortisol, hyperglycemia, and hypokalemia. Aldosterone was also above the normal limit, potentially suggesting autonomous co-secretion, though formal aldosterone-suppression testing was not performed (Table 1).

**Table 1:** Biochemistry and hormonal tests of patient number 1 during the first hospitalization

	Conventional (range) SI (range)	March 31, 2023	April 4, 20/23	April 7, 2023	April 11, 2023	April 17, 2023	April 24, 2023
Cortisol	μg/dL (4.8-19.5)	42.4 μg/dL	29.8 μg/dL		17.8 μg/dL	10.9 μg/dL	
	nmol/L (132.4-537.9)	1169.6 nmol/L	822.0 nmol/L		491.0 nmol/L	300.7 nmol/L	
Urinary free cortisol	μg/24 hours (5.8-61.6)			26.5 μg/24h			
	nmol/24 hours (16-170)			73 nmol/24h			
ACTH	pg/mL (7-63)	<3 pg/mL			9 pg/mL		9 pg/mL
	pmol/L (1.54-13.87)	<0.66 pmol/L			1.98 pmol/L		1.98 pmol/L
DHEAS	μg/dL (35-430)	465 μg/dL					35 μg/dL
	μmol/L (0.95-11.67)	12.62μmol/L					0.95 μmol/L
Testosterone	ng/dL (3-41)	197 ng/dL			76 ng/dL		58 ng/dL
	nmol/L (0.10-1.42)	6.83 nmol/L			2.63 nmol/L		2.01 nmol/L
Estradiol	pg/mL (<138)	45 pg/mL					
	pmol/L (<506.6)	165.2 pmol/L					
Aldosterone	ng/dL (5-25)	67 ng/dL			29 ng/dL	17 ng/dL	
	nmol/L (1.39-0.69)	1.86 nmol/L			0.80 nmol/L	0.47 nmol/L	
Potassium	mmol/L (3.4-4.5)	2.3 mmol/L	2.5 mmol/L	3.2 mmol/L	4.3 mmol/L	4 mmol/L	3.9 mmol/L
Sodium	mmol/L (136-145)	141 mmol/L	143 mmol/L	140 mmol/L	139 mmol/L	146 mmol/L	145 mmol/L
Glucose	mg/dL (76-115)	183 mg/dL	144 mg/dL	154 mg/dL	106 mg/dL	103 mg/dL	116 mg/dL
	mmol/L (4.22-6.38)	10.16 mmol/L	7.99 mmol/L	8.55 mmol/L	5.88 mmol/L	5.72 mmol/L	6.44 mmol/L

Abbreviation: DHEAS, dehydroepiandrosterone sulfate.

### Case 2

In October 2024, he experienced severe exacerbation of Cushing syndrome, and CT documented bilateral lung lesions, multiple liver nodules, and disease relapse. He was therefore referred to our center.

## Treatment

### Case 1

Intravenous potassium, subcutaneous insulin, and high-dose oral osilodrostat (30 mg/day in 2 administrations) were started with excellent tolerance. As symptoms improved, with concomitant normalization of cortisol, testosterone, dehydroepiandrosterone sulfate (Table 1), potassium, and glucose plasma levels, oral mitotane treatment was started at 1.5 g/day (3 times a day) 10 days after admission; antihypertensive therapy was deescalated and insulin discontinued within 20 days.

Low-dose single-agent intravenous cisplatin (40 mg) was administered on the 18th day after admission, as full-dose combination chemotherapy was considered unsafe given the patient's poor clinical condition at presentation. She was then discharged the following week on osilodrostat 20 mg/day, mitotane 2.5 g/day, and cortisone acetate 37.5 mg/day (2 times a day).

Before readmission for the second chemotherapy cycle, due on May 4, 2023, the patient was admitted to another hospital on April 28, 14 days after discharge, due to pneumococcal pneumonia. Mitotane was suspended while osilodrostat was maintained throughout. She was discharged after 15 days with progressively improving clinical conditions; osilodrostat was continued, but mitotane was not reintroduced.

**Figure 1:**
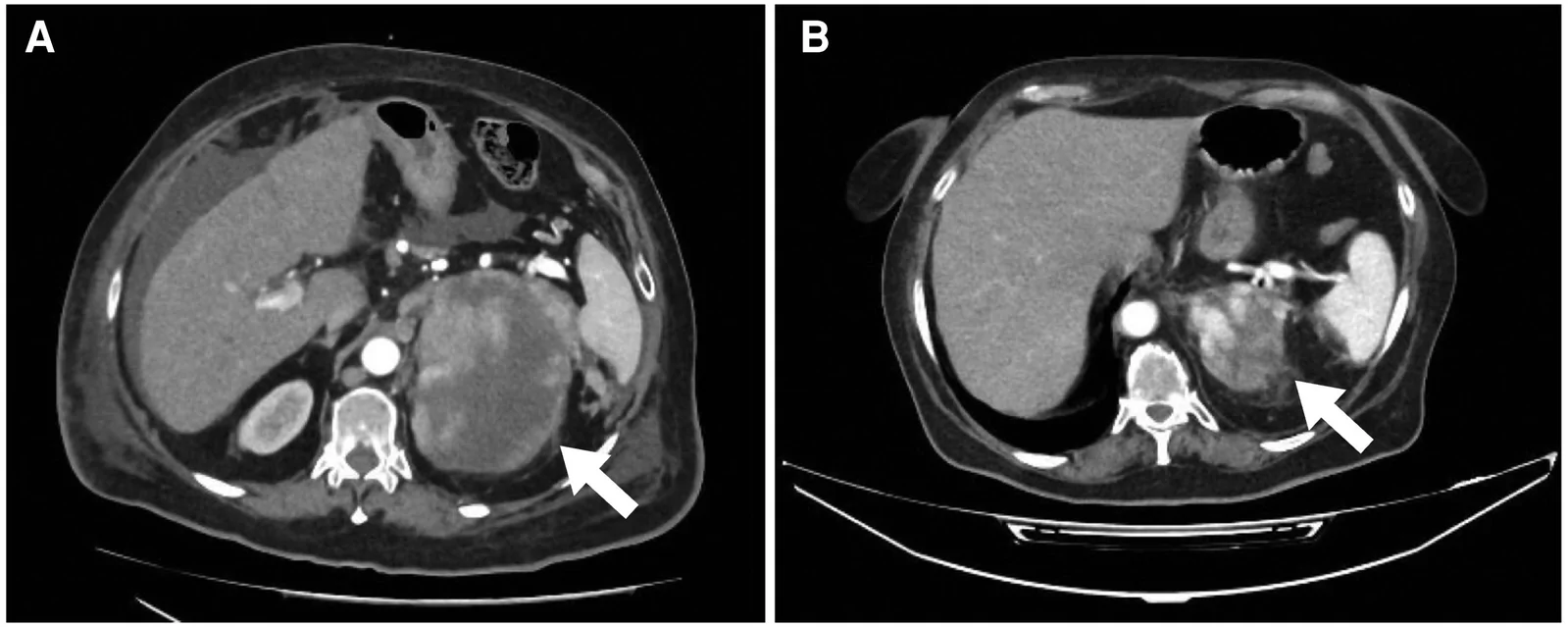
Comparison of left adrenal lesion size between the computed tomography scan of April 2023 (A) and the computed tomography scan from June 2023 (B).

On readmission to our institution on June 19, 2023, 42 days after discharge, the patient was in good clinical condition (ECOG PS 1) and fully autonomous in the activities of daily living. A restaging CT documented a substantial reduction of the left adrenal mass (70 × 60 mm); abdominal adenopathies also significantly decreased both in size and number (Fig. 1), despite mitotane levels of 5.10 mg/L. Osilodrostat was progressively reduced and discontinued at discharge on the fifth day postadmission. Intravenous cisplatin was continued for 4 additional cycles.

After a total of 5 chemotherapy cycles, with target mitotane concentrations (15.80 mg/L) reached 4 months after initiation, a new CT revealed a further reduction of the left adrenal lesion (40 × 57 mm), while abdominal lymphadenopathies were stable. The patient was referred to surgery, and maintenance mitotane monotherapy was introduced.

### Case 2

At presentation, he was in poor clinical condition (ECOG PS 3), with marked lower-limb edema and peripheral neuropathy limiting autonomous ambulation. Total serum cortisol was modest, probably reflecting hypoproteinemia with underestimation of true cortisol excess. Oral mitotane monotherapy (2 g/day 3 times a day) was started with oral metyrapone (750 mg/day in 3 administrations); chemotherapy was omitted. Cortisol excess symptoms improved rapidly. Four days later, due to hypocortisolism symptoms despite oral cortisone acetate 37.5 mg/day in 2 administrations, metyrapone was reduced to 500 mg/day. He was discharged after 24 days. In December 2024, he was admitted elsewhere for syncope and exertional dyspnea due to bilateral pneumonia. Mitotane was interrupted and intravenous antibiotics administered, while metyrapone was continued throughout. At discharge, mitotane plasma concentration reached 2.54 mg/L.

In January 2025, the patient presented for outpatient evaluation. The paitent’s general condition had significantly improved, with progressive weight loss from 119 to 110 kg, reduction of bilateral lower-limb edema, and improved blood pressure control. Blood tests showed electrolytes within limits and cortisol in the normal range (Table 2).

**Table 2:** Biochemistry and hormonal tests of patient number 2 during the first hospitalization and afterwards

		During hospitalization					
	Conventional (range) SI (range)	October 16, 2024	October 28, 2024	November 4, 2024	February 6, 2025	May 20, 2025	December 28, 2025
Cortisol	μg/dL (4.8-19.5)	15.2 μg/dL		9.2 μg/dL	18.7 μg/dL	6.1 μg/dL	10.8 μg/dL
	nmol/L (132.4-537.9)	419.3 nmol/L		253.8 nmol/L	515.9 nmol/L	168.3 nmol/L	297.9 nmol/L
Urinary free cortisol	μg/24 hours (5.8-61.6)	127.8 μg/24h					
	nmol/24 hours (16-170)	353 nmol/24h					
ACTH	pg/mL (7-63)	<3 pg/mL			3.2 pg/mL	6.2 pg/mL	5.8 pg/mL
	pmol/L (1.54-13.87)	<0.66 pmol/L			0.70 pmol/L	1.37 pmol/L	1.28 pmol/L
DHEAS	μg/dL (35-430)	62 μg/dL			25 μg/dL		14 μg/dL
	μmol/L (0.95-11.67)	1.68 μmol/L			0.68 μmol/L		0.38 μmol/L
Testosterone	ng/dL (193-740)	340 ng/dL			610 ng/dL		580 ng/dL
	nmol/L (6.7-25.7)	11.8 nmol/L			21.1 nmol/L		20.1 nmol/L
Estradiol	pg/mL (<138)	<25 pg/mL			16 pg/mL		
	pmol/L (<506.6)	<91.8 pmol/L			58.7 pmol/L		
Aldosterone	ng/dL (5-25)	19.6 ng/dL			3.7 ng/dL		
	nmol/L (1.39-0.69)	0.543 nmol/L			0.103 nmol/L		
Potassium	mmol/L (3.4-4.5)	4.8 mmol/L	5 mmol/L	3.9 mmol/L	4.4 mmol/L		3.8 mmol/L
Sodium	mmol/L (136-145)	140 mmol/L	138 mmol/L	142 mmol/L	142 mmol/L		144 mmol/L
Glucose	mg/dL (76-115)	84 mg/dL	88 mg/dL	67 mg/dL	75 mg/dL		71 mg/dL
	mmol/L (4.22-6.38)	4.66 mmol/L	4.88 mmol/L	3.72 mmol/L	4.16 mmol/L		3.94 mmol/L

Abbreviations: DHEAS, dehydroepiandrosterone sulfate.

**Figure 2:**
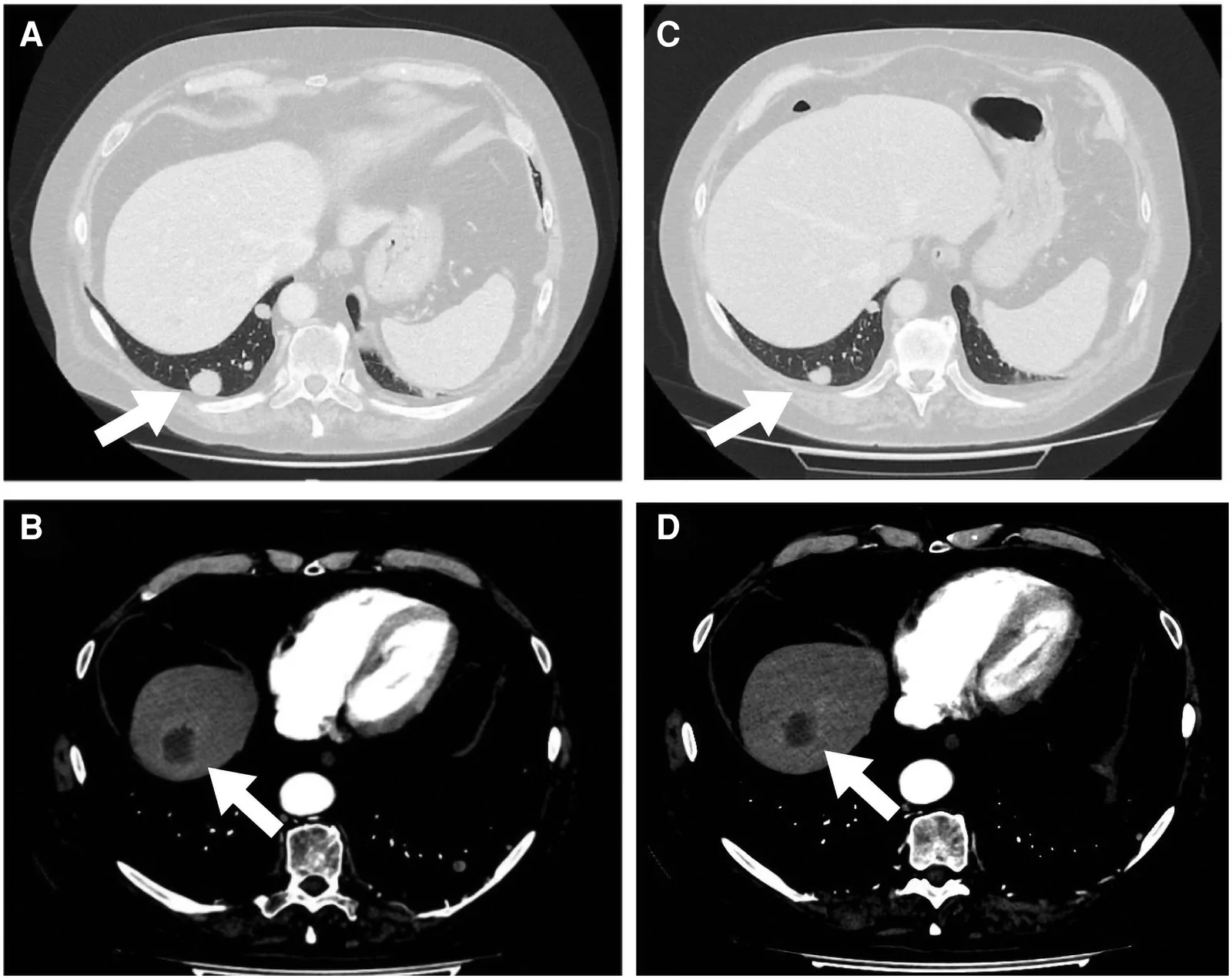
Comparison of lung and liver metastasis between the computed tomography scan of October 2024 (A and B) and the computed tomography scan of February 2025 (C and D).

A restaging CT performed 5 days later showed stable disease with a slight size decrease of the known liver, lung, and adrenal lodge lesions (Fig. 2). Mitotane serum level was 3.8 mg/L. Oral mitotane was resumed at 3 g/day while maintaining metyrapone and cortisone acetate 25 mg/day.

On May 5, 2025, after 3 months, clinical condition had improved, body weight remained stationary, and plasma mitotane concentration reached 9.24 mg/L. Blood tests showed low morning cortisol and suppressed ACTH (Table 2). A restaging CT documented a significant disease response, with reduction in size of both lung and liver nodules.

## Outcome and follow-up

### Case 1

In March 2024, after a disease-free interval of 5.5 months, relapse in lung, lymph node, liver, and peritoneum was documented while cortisol remained suppressed. A rechallenge with intravenous continuous-infusion cisplatin was started but, after only 2 months, a subsequent CT documented progression in all sites. Due to worsening clinical condition, no further oncological treatment was initiated, and the patient was referred to best supportive care until death, which occurred on December 20, 2024, 22 months after diagnosis.

### Case 2

Over the next 6 months, clinical conditions remained stable, with further weight loss (98 kg) and improvement in lower-limb edema. Oral metyrapone was progressively reduced and then interrupted after 8 months on September 4, 2025. Blood pressure control remained excellent, and blood tests showed suppressed plasma cortisol (Table 2). Plasma mitotane reached the therapeutic range at 16.71 mg/L. The subsequent CT scans, performed in August and December 2025, documented a dramatic numerical and dimensional reduction of lung, liver, and abdominal nodules.

## Discussion

Management of metastatic ACC remains extremely challenging, particularly with overt Cushing syndrome. Here, controlling cortisol hypersecretion is a priority, and the recommendation is to “hit hard and fast,” as the syndrome causes severe morbidity and mortality and contraindicates chemotherapy [17].

We describe 2 patients with metastatic ACC and severe Cushing syndrome treated with block-and-replace using osilodrostat or metyrapone. In both, cortisol normalized rapidly and safely, with marked clinical improvement. Although Pneumocystis jirovecii pneumonia prophylaxis was not administered in these cases, rapid correction of severe hypercortisolism should be accompanied by trimethoprim-sulfamethoxazole prophylaxis, given the immunosuppression associated with cortisol excess and the risk of immune reconstitution on rapid reversal [18]. Notably, both patients experienced an unexpected radiological tumor response far before therapeutic mitotane plasma concentrations and without effective cytotoxic chemotherapy. This raises the hypothesis that steroidogenesis inhibition itself may exert a direct or indirect antineoplastic effect [19].

In the first case, the dramatic reduction in tumor burden at first restaging cannot reasonably be attributed to chemotherapy, as the patient received only a single low-dose cisplatin administration. Mitotane exposure was likewise short and well below the therapeutic range, making a mitotane-driven effect unlikely. Large retrospective series show that mitotane monotherapy is rarely effective in synchronous metastatic disease with high tumor burden, as in this case [20]. Importantly, osilodrostat was the only systemic therapy continuously administered throughout the clinical course preceding first re-evaluation, including during hospitalization for severe pneumonia, strongly supporting a potential role of steroidogenesis inhibition in driving tumor response.

Similarly, in the second case, disease stabilization and subsequent tumor shrinkage were observed while mitotane plasma concentrations remained far below the therapeutic threshold. Dose escalation was possible only later, after clinical improvement. The early radiological response therefore occurred in a phase during which metyrapone represented the only biologically active treatment.

Several mechanisms may explain these findings. Glucocorticoids can promote tumor progression through multiple pathways, including immunosuppression, inhibition of apoptosis, and stimulation of tumor cell survival signaling [5, 21, 22]. Excess cortisol may also impair antitumor immune surveillance by suppressing T-cell activation and natural killer cell function. Rapid cortisol blockade could therefore restore immune competence and favor tumor control [21, 23].

Moreover, preclinical evidence suggests that inhibiting steroidogenic enzymes may be cytotoxic to ACC cells. Abiraterone, a CYP17 inhibitor, produced growth inhibition in ACC cell lines and xenograft models [24], supporting that disruption of intratumoral steroidogenesis can impair tumor viability. Although osilodrostat and metyrapone target different enzymes, a similar mechanism cannot be excluded and deserves further investigation.

Block-and-replace therapy may thus offer dual benefits in ACC with Cushing syndrome: rapid clinical stabilization and potential tumor growth suppression. While mitotane and etoposide, doxorubicin, and cisplatin–based chemotherapy remain the backbone of systemic therapy, early aggressive control of hypercortisolism may improve treatment feasibility and directly contribute to disease control.

We acknowledge the intrinsic limitations of a case report and that mitotane levels below the conventional therapeutic range (14-20 mg/L) may still contribute meaningfully to antitumor activity [25]. Nevertheless, the temporal relationship between steroidogenesis blockade and tumor response in 2 independent cases is striking and biologically plausible.

In conclusion, these cases suggest a potential antineoplastic effect of steroidogenesis inhibitors within a block-and-replace strategy in patients with ACC-related Cushing syndrome. These hypothesis-generating observations remain speculative and deserve further biomarker and mechanistic investigations.

## Learning points

- Rapid control of hypercortisolism is essential in metastatic ACC with Cushing syndrome; a block-and-replace strategy with steroidogenesis inhibitors can normalize cortisol and markedly improve clinical status.
- Steroidogenesis inhibition may contribute to early tumor control, as radiological responses occurred before therapeutic mitotane levels and without effective chemotherapy.
- Blocking cortisol excess may exert indirect or direct antitumor effects, potentially by restoring antitumor immune function and disrupting tumor steroidogenic pathways.

## Contributors

All authors made individual contributions to authorship. A.B., D.C., M.L., and A.T. were responsible for the clinical management of both patients during the hospital admissions described in this report. A.B., D.C., M.L., A.T., S.R., and D.L.B. were involved in the patients' subsequent evaluation during outpatient follow-up visits and during subsequent lines of therapy. D.L.B. was responsible for writing the manuscript and original draft preparation. D.L.B., S.R., and A.B. were responsible for reviewing and editing. A.B., D.C., M.L., and A.T. were responsible for validation and supervision. All authors have read and agreed to the published version of the manuscript.

## Data Availability

Data sharing is not applicable to this article as no datasets were generated or analyzed during the current study.
